# Effect of dietary Chinese herbal preparation on dry matter intake, milk yield and milk composition, serum biochemistry, hematological profile, and reproductive efficiency of Holstein dairy cows in early postpartum period

**DOI:** 10.3389/fvets.2024.1434548

**Published:** 2024-07-19

**Authors:** Adili Abulaiti, Umair Ahsan, Zahid Naseer, Zulfiqar Ahmed, Wenju Liu, Chongmei Ruan, Xunsheng Pang, Shujuan Wang

**Affiliations:** ^1^College of Animal Science, Anhui Science and Technology University, Fengyang, Anhui, China; ^2^Anhui Province Key Laboratory of Animal Nutritional Regulation and Health, Fengyang, Anhui, China; ^3^Department of Plant and Animal Production, Burdur Vocational School of Food, Agriculture and Livestock, Burdur Mehmet Akif Ersoy University, İstiklal Yerleşkesi, Burdur, Türkiye; ^4^Center for Agriculture, Livestock and Food Research, Burdur Mehmet Akif Ersoy University, İstiklal Yerleşkesi, Burdur, Türkiye; ^5^Faculty of Veterinary and Animal Sciences, Pir Mehr Ali Shah Arid Agriculture University, Rawalpindi, Pakistan; ^6^Faculty of Veterinary and Animal Sciences, University of Poonch, Rawalakot, Azad Jammu and Kashmir, Pakistan; ^7^College of Life and Health Science, Anhui Science and Technology University, Fengyang, Anhui, China

**Keywords:** Holstein cows, Chinese herbal medicine, modified GPGMH Ovsynch synchronization, milk production, reproductive performance

## Abstract

The present study investigated the effects of various inclusion levels of dietary Chinese herbal medicine (CHM) preparation on feed consumption, milk yield and milk composition, serum biochemistry, hematological profile, and reproductive efficiency of Holstein dairy cows. A total of 117 lactating Holstein cows were randomly divided into four groups as control (*n* = 27; without CHM supplementation) and treatment groups CHM-0.5 (*n* = 31), CHM-0.75 (*n* = 29), and CHM-1 (*n* = 30) fed diet supplemented with 0.5, 0.75, and 1 kg/cow/d for 30 days, respectively. The study began at d 20 postpartum (d 0 of the study). At d 50 postpartum, the cows in all groups were subjected to estrus synchronization using a modified Ovsynch protocol (GPGMH) and observed for reproductive variables. Feed intake, milk yield and milk composition, serum biochemistry and hematological profile, and reproductive efficiency were measured. A significantly higher milk yield with improved milk lactose, milk protein and milk fat were found in the CHM-0.75 group compared to the other groups (*p* < 0.05). Besides, the estrus response, ovulation rate, ovulatory follicle diameter, and pregnancy rate increased in CHM-0.75 compared to CHM-0 or CHM-0.5 group (*p* < 0.05). The serum metabolites (glucose, AST, arginine, BUN, and NO) showed variations among the treatment groups at different time points (synchronization, AI, or post-AI). In conclusion, CHM supplementation improves the milk yield, milk composition, and serum metabolites in dairy cows. Daily supplementation of 0.75 kg CHM before the GPGMH protocol application enhances the reproductive traits in dairy cows under summer conditions.

## 1 Introduction

In recent years, animal production in China has developed rapidly and it has become a key component of the agriculture sector. High-density large-scale commercial farming and intensive breeding paradigm lead to the deterioration of animal physiology. For efficient growth and maintenance of productivity, feed antibiotics were a common practice for the prevention and cure of animal diseases. The excessive use of antibiotics in livestock production amplified the antimicrobial resistance, high antibiotics residues in animal products and environmental contamination that negatively affected public health (1). Consequently, the use of in-feed antibiotics was banned in the European Union in 2006 followed by further ban in different countries across the globe. This attracted the development of alternatives for use in animal diets. In this scenario, Chinese herbal medicine (CHM) preparations have received considerable attention in recent years than synthetic feed additives. Due to the versatile composition of CHM preparations, several attempts have been made to replace antibiotics. The CHM preparations contain polysaccharides, vitamins, alkaloids, amino acids, saponins and act as immune enhancers, anti-stress agents, and hormone-like agents, insect repellents, galactagogues, and disease control agents (2). Previous studies have documented the beneficial effects of dietary CHM on growth, feed utilization, reproductive performance, and quality of animal products (3).

These preparations can boost anabolism and digestion by increasing the absorption of nutrients during the growth phase. Dietary supplementation of calf starter diet with CHM (four main Chinese herbs, i.e., *Leonurus cardiaca*, *Taraxacum officinale*, *Ligustrum lucidum*, and *Hordeum vulgare*) enhanced the rumen environment and improve the daily weight gains of calves (4). Similarly, piglets fed traditional CHM synergized with probiotics in grower-finisher ration had greater average daily gain (5). The CHM not only improve the growth but also enhance the reproductive efficiency of dairy cows (6) and sows (7). The use of other herbs, such as *Agaricus bisporus*, *Acorus tatarinowii*, *Wolfiporia extensa*, *Bupleurum falcatum*, *Crataegus monogyna Jacq.,* and *Agastache rugosa* also improved the feed intake in cows (8, 9). The ingestion of *Crataegus monogyna* and dried tangerine peel is greater in cows due to its attractive aroma. Additionally, the growth and development of mammary glands, increment in hormones levels, and improved lactation were significant outcomes of feeding CHM as supplements (10). A formulation with *Glycyrrhiza glabra, Astragalus L. (Fabaceae),* and *Gardenia jasminoides* successfully cured the subclinical mastitis in cows (11).

Keeping in view the beneficial effects of different CHM on cow health and production (12), we hypothesized that the inclusion of CHM in diets for dairy cows in early postpartum period might be useful to improve the growth, milk yield, and reproductive efficiency of dairy cows by improving the feed intake, digestibility, and nutrients absorption. Therefore, the present study was designed to evaluate the effects of a CHM preparation on the uterine involution rate, dry matter intake and blood metabolites, milk yield and composition, and reproductive performance of lactating Holstein cows in early postpartum period.

## 2 Materials and methods

Before execution of the study, an approval was obtained from the Animal Experimental Ethical Inspection of Laboratory Animal Centre, Huazhong Agricultural University, Wuhan (HZAUCA-2018-004). All the experimental protocols were performed according to the guidelines of the Committee of Animal Research Institute of the university.

### 2.1 Location and climatic conditions

The study was conducted during the summer season (July 12, 2022, to August 30, 2022) at Hebei JunYing dairy farm Co., Ltd. Hebei province (30°32′N, 111°5′E), China. The farm is geographically located in a subtropical monsoon region with average temperatures ranging from 1 to 5°C in winter and 27 to 35°C during summer (reaching up to 40°C sometimes in July and August months). During the study period, the average temperature was 36.5 ± 0.2°C, relative humidity 79.0 ± 1.1%, and temperature-humidity index (THI) was 91.0 ± 0.3. The observed THI level showed intense heat stress for dairy cows maintained in Hubei province.

### 2.2 Chinese herbal medicine

The CHM used in this study was based on Shi-Quan Da Bu Decoction procedures (13). The CHM contained the extracts of plants (*Eucommia ulmoides*, *A. L.* (Fabaceae), *Codonopsis pilosula*, *Angelica archangelica*, Atractylodes amurensis *G. glabra*, *Ligusticum striatum*, *W. extensa*, and *Pueraria montana*) extracted twice in the aqueous medium. The extracts were subjected to fermentation using *Lactobacillus acidophilus* (0.2 × 10^8^ CFU/g), *Lactobacillus plantarum* (0.2 × 10^8^ CFU/g), *Bacillus subtilis* (0.2 × 10^8^ CFU/g), *Bacillus coagulans* (0.2 × 10^8^ CFU/g), *Bacillus licheniformis* (0.2 × 10^8^ CFU/g), *Enterococcus faecalis* (0.2 × 10^8^ CFU/g), and *Saccharomyces cerevisiae* (0.2 × 10^8^ CFU/g). All these ingredients were fermented (anaerobic) followed by cold granulation to prepare the fermented granules of CHM additive (Cat. C6H10N205, Longxi Huachuang Biotechnology Co. Ltd. Dingxi, Gansu province, China) used in the present study. The CHM preparation was characterized by total flavonoids, polyphenols, glycyrrhetinic acid, saponins, polysaccharides, and acids. Detailed nutritional composition and physicochemical properties of CHM have been presented in Table 1.

**Table 1:** Chemical composition and physicochemical properties of Chinese herbal medicine used in the study.

Item	Description	Item	Concentration
Color	Dark brown	Flavonoids, mg/kg	500
Odor	Medicinal aroma	Polyphenols, mg/kg	500
Total viable bacteria	5.4 × 10^8^ cfu/g	Carotenoids, mg/kg	50
Aflatoxin B1	Not detected	Polysaccharides, g/kg	20
*Escherichia coli*	Not detected	Total acids, g/kg	38
Salmonella	Not detected	Saponins, g/kg	17
Lead	Not detected	Glycyrrhetinic acid, g/kg	2
Fluoride, mg/kg	12	Moisture, %	39.2
Arsenic, mg/kg	0.419	Ash, %	4.2
Mercury, mg/kg	0.036	Crude protein, %	8.6
Lactic acid bacteria, cfu/g	1.3 × 10^7^	Crude fiber, %	25.9
Total mold count, cfu/g	1.8 × 10^3^	Starch, %	2.18
		Crude fat, %	2.4

### 2.3 Study design

A total of one hundred and seventeen (*n* = 117) multiparous Holstein dairy cows in early postpartum period, aged between 4 and 7 years, were randomly divided into four experimental groups 20 days after parturition, i.e., control/CHM-0 (*n* = 27; without CHM supplementation), CHM-0.5 (*n* = 31; CHM 0.5 kg/cow/d), CHM-0.75 (*n* = 29; CHM 0.75 kg/cow/d), and CHM-1 (*n* = 30; CHM 1 kg/cow/d). Cows used in this study had moderate body condition score (3.4 ± 0.6) with average body weight of 578.1 ± 86.5 kg. The measured daily dose of CHM for each respective group was first sprinkled over concentrate, homogeneously mixed in total mixed ration (TMR) wagon with other ingredients and offered to the cows. Dietary supplementation of CHM was carried out twice a day. Measured daily dose of each group was divided into two and fed after mixing in morning and evening TMR meals. After each meal, it was ensured that each cow had consumed the approximate dose of CHM properly. The cows were supplemented with CHM for 30 days starting from d 20 of calving (d 0 of the study).

### 2.4 Management conditions

Cows were housed in a semi-intensive shed with cemented rooftop and the shed was fenced by galvanized wire mesh on two sides. Ventilation and cooling in the summer was provided by two exhaust fans and sprinklers installed inside the shed. Cows were fed a TMR twice a day (morning and evening) formulated according to the nutrient requirements of dairy cows (Table 2) recommended by NRC (2001) in an open feeding system. Cows had *ad libitum* access to fresh and clean water.

**Table 2:** Composition of basal total mixed ration fed to the Holstein dairy cows (as fed basis).

Item	%
Corn silage	48.02
Concentrate	24.98
Alfalfa	10.19
Corn, flaked	4.68
Oats, flaked	3.24
Whole cottonseed	3.60
Sugar beet pulp, pelleted	3.60
Soybean, puffed	0.96
Rumen bypass fat	0.36
Probiotics	0.19
Rumen protected glucose	0.12
Sodium bicarbonate	0.06
Nutritional composition (%, dry matter basis)	
Net energy for lactation, MJ/kg	7.21
Crude protein	13.81
Crude fat	4.49
Neutral detergent fiber	31.08
Phosphorus	0.31
Calcium	0.40

### 2.5 Dry matter intake

Dry matter intake (DMI) of cows was measured based on the feed consumption computed from the difference of amounts of daily feed offered and orts each day. Feed not consumed was weighed before the morning feeding. The DMI was calculated using the dry matter content of the diet and amount of feed consumed.

### 2.6 Fecal composition

At d 0, d 15, and d 30, cows (*n* = 8) were randomly selected from each group and feces were collected directly from the rectum of the cows and analyzed for dry matter (DM), crude protein (CP), acid detergent fiber (ADF), neutral detergent fiber (NDF), lignin, starch, total fatty acids (TFA), crude ash, Ca, P, Mg, and K using methods previously described in detail by Association of Official Analytical Chemists (14).

### 2.7 Milk yield and milk composition

Cows were milked thrice a day (at 06:00, 14:00, and 20:00) by machine milking throughout the supplementation period and the milk yield was recorded using carefully calibrated jars. Milk composition was analyzed on d 0, d 15 and d 30 of CHM supplementation using milk analyzer (MilkoScan Type 78,110; Foss A/S, Hillerød, Denmark).

### 2.8 Serum biochemistry, immunoglobulins, and hematological profile

Blood samples were randomly collected from the coccygeal veins of 5 cows in each group on d 0, d 15 and d 30 of CHM supplementation to evaluate the serum biochemistry (without anticoagulant) and hematologic profile (with anticoagulant) of dairy cows. Serum was separated by centrifugation after the clotting and subjected to analysis of alanine aminotransferase (ALT), aspartate aminotransferase (AST), total protein (TP), albumin (ALB), globulin (GLB), alkaline phosphatase (ALP), γ-glutamyl transpeptidase (GGT), lactate dehydrogenase (LDH), total bilirubin (TBIL), creatine kinase (CK), amylase, total bile acid (TBA), glucose (GLU), total cholesterol (CHOL), uric acid (UA), blood urea nitrogen (BUN), creatinine (CREA), triglyceride (TG), potassium (K), sodium (Na), chloride (Cl), calcium (Ca), phosphorus (P), and magnesium (Mg) using commercial kits (Beijing Beijing Xinchuangyuan Biotechnology Co. Ltd., China) followed by reading in an automatic biochemical analyzer (TBA-120FR Auto Clinical Chemistry Analyzer, Toshiba Corporation, Tokyo, Japan).

Serum immunoglobulins A (IgA), G, (IgG), and M (IgM) were quantified by single radial immune diffusion method using commercially available kits for IgA, IgG, and IgM (Beijing North Biotechnology Research Institute Co., Ltd., Beijing, China).

Blood samples were subjected to automatic blood analyzer (Beckman Coulter Blood Routine Analyzer, Beckman Coulter Trading Co. Ltd. China) for hematological profile of dairy cows in terms of complete blood count.

### 2.9 Application of modified ovarian synchronization protocol

**Figure 1:**
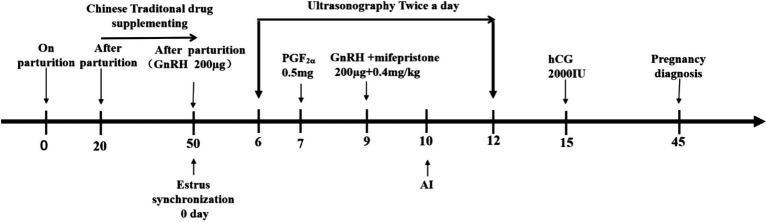
Timeline of the Chinese herbal medicine supplementation and the modified Ovsynch synchronization protocol.

After 50 days of calving, a modified Ovsynch protocol (GPGMH) was used to synchronize the cows (15). Briefly, the first injection of gonadotropin-releasing hormone (GnRH 200 μg, Ningbo Sansheng Pharmaceutical Industry Co., Ltd., Ningbo, Zhejiang, China) was given to all the cows on day 0, an injection of PGF2α (0.5 mg, Ningbo Sansheng Pharmaceutical Industry Co., Ltd., Ningbo, Zhejiang, China) on day 7 of protocol and second dose of GnRH (200 μg) and mifepristone (0.4 mg/kg, IM, Hubei Yun Cheng Sai Technology, China) day 9 of protocol. The cows were inseminated artificially (AI) within 24 h of the 2nd GnRH dose. Moreover, the estrus signs (vaginal mucous discharge, bellowing, excitation, swollen vagina, and mounting behavior) were observed physically twice daily. An injection of human chorionic gonadotropin (hCG 2000 IU; Ningbo Sansheng Pharmaceutical Industry Co., Ltd., Ningbo, Zhejiang, China) was also administered to each cow on day 5 of AI (Figure 1).

Ultrasound scanner (WED-9618-v, Shenzhen Well.D Medical Electronics Co. Ltd., Guangdong, China) equipped with rectal probe (LV2-3/6.5 MHz) was used to monitor follicular dynamics twice daily (between 6 to 12th day of protocol). Ovulation was recorded by the sudden disappearance of dominant follicles between subsequent ultrasonographic examinations. Pregnancy diagnosis was made rectally by ultrasound scanner on the 35th day after AI, confirming fetal presence and viability.

### 2.10 Evaluation of uterine involution rate

Sixty-two postpartum cows (Among them, 12 cows were suffereing from uterine infections and 50 cows were uterine diseases) were monitored for uterine recovery during the CHM supplementation time. The rectal ultrasonography of the uterus was undertaken with a B-mode veterinary ultrasound scanner (LV2-2/6.5 MHZ, linear array transducer, Shenzhen, China). Photographs of the uterus were transferred to a computer workstation for analysis to assess the extent of uterine involution at different postpartum days according to the method established in our laboratory (16). Before the examination, the feces were gently removed from the rectum, the probe was inserted, and placed on the uterus. The same operator performed all ultrasound examinations to avoid system errors.

### 2.11 Statistical analysis

All the data were tested for normality using Shapiro–Wilk’s test. Non-normalized variables were transformed by logarithmic or square-root transformation for normalization. The data related to DMI, fecal composition, body weight gain of calves, milk yield and milk composition, serum biochemistry, hematological profile, and reproductive traits were subjected to one-way analysis of variance (ANOVA) followed by Duncan’s multiple range test as post-hoc test. Chi-square test was applied to analyze the data of estrus, ovulation, and pregnancy. Differences among the groups were assumed significant at 95% confidence interval (*p* < 0.05). All the statistical analyses were applied in a statistical package SPSS (version 24.0; IBM Corp., Armonk, NY, United States). Results were presented as mean ± standard deviation.

## 3 Results

### 3.1 Dry matter intake and fecal composition

The DMI of Holstein dairy cows fed different levels of dietary CHM has been shown in Table 3. The DMI was greater in CHM-0.5 and CHM-0.75 groups at d 0, CHM-0.75 at d 7, d 28, and d 36 compared to other dietary treatments (*p* < 0.05). In general, DMI increased in CHM-0.5 and CHM-0.75 groups whereas, no significant change in DMI was seen in other groups.

**Table 3:** Dry matter intake (kg) of Holstein dairy cows fed different levels of dietary Chinese herbal medicine in early postpartum period.

Day	Control	CHM-0.5^1^	CHM-0.75^1^	CHM-1^1^
7	21.2 ± 0.7^Bab^	19.8 ± 2.1^Bb^	22.2 ± 0.9^Ba^	21.5 ± 0.3^ab^
14	21.8 ± 0.4^B^	21.1 ± 1.9^AB^	22.4 ± 0.5^B^	21.9 ± 0.9
28	22.3 ± 0.4^Aab^	21.4 ± 0.6^ABb^	23.6 ± 1.0^Aa^	21.8 ± 0.4^b^
36	22.6 ± 0.3^Ab^	22.2 ± 0.7^Ab^	23.5 ± 0.2^Aa^	22.1 ± 0.3^b^

^a,b^Means with different superscripts in the same row are significantly different (*p* < 0.05). ^A,B^Means with different superscripts in the same column against the same variable are significantly different (*p* < 0.05). ^1^CHM-0.5 = 0.5 kg CHM; CHM-0.75 = 0.75 kg CHM; CHM-1 = 1 kg CHM.

Table 4 shows the fecal composition of dairy cows fed diets supplemented with different levels of dietary CHM. Fecal composition of Holstein dairy cows in early postpartum period remained unaffected across the groups except crude ash which was greater in CHM-0.75 group than CHM-1 group at d 15 (*p* < 0.05).

**Table 4:** Fecal composition of Holstein dairy cows fed different levels of dietary Chinese herbal medicine in early postpartum period.

Item	Day	Control	CHM-0.5^1^	CHM-0.75^1^	CHM-1^1^
Moisture, %	0	82.1 ± 0.8	81.9 ± 0.5	84.9 ± 0.7	85.2 ± 1.6
	15	86.1 ± 0.9	83.9 ± 2.4	85.7 ± 1.8	86.2 ± 1.5
	30	85.3 ± 1.3	84.9 ± 2.1	85.4 ± 1.7	82.9 ± 4.7
Crude protein, g/kg	0	15.4 ± 2.6	15.1 ± 2.2	15.9 ± 1.2	14.75 ± 1.1
	15	17.7 ± 2.4	17.0 ± 1.9	17.3 ± 1.9	17.0 ± 1.1
	30	16.2 ± 1.8	16.5 ± 4.9	15.1 ± 1.7	16.3 ± 2.5
Acid detergent fiber, g/kg	0	38.9 ± 2.8	38.1 ± 3.3	39.1 ± 2.4	37.5 ± 4.1
	15	40.6 ± 2.5	41.4 ± 4.2	41.5 ± 3.8	38.9 ± 3.7
	30	39.9 ± 3.2	39.7 ± 6.7	40.4 ± 2.4	38.3 ± 5.0
Neutral detergent fiber, g/kg	0	55.4 ± 4.8	54.3 ± 4.6	53.9 ± 2.9	52.2 ± 4.5
	15	56.8 ± 4.1	56.2 ± 4.5	56.2 ± 5.6	56.0 ± 3.9
	30	56.9 ± 5.0	55.9 ± 10.1	57.6 ± 2.3	52.9 ± 5.2
Lignin, g/kg	0	9.1 ± 0.6	9.4 ± 1.1	9.9 ± 1.1	9.1 ± 1.5
	15	10.9 ± 0.6	10.6 ± 1.1	11.3 ± 1.3	10.3 ± 1.3
	30	9.9 ± 0.7	9.6 ± 1.9	9.8 ± 0.9	10.0 ± 1.1
NDF after 240 h, g/kg	0	47.5 ± 2.1	49.2 ± 4.9^B^	47.2 ± 5.4	47.4 ± 5.9^B^
	15	48.8 ± 2.3	50.9 ± 4.6^B^	48.0 ± 5.0	45.8 ± 6.1^B^
	30	51.4 ± 1.1	57.0 ± 7.7^A^	51.9 ± 2.9	54.6 ± 6.4^A^
Starch, g/kg	0	1.5 ± 0.6^B^	2.5 ± 0.6	2.0 ± 1.2^B^	1.6 ± 0.7^B^
	15	1.6 ± 0.5^B^	2.5 ± 0.5	1.9 ± 1.1^B^	1.8 ± 0.7^B^
	30	3.3 ± 1.9^A^	2.8 ± 1.7	3.3 ± 0.6^A^	4.5 ± 3.2^A^
Total fatty acids, g/kg	0	2.1 ± 0.2	2.4 ± 0.8	2.3 ± 0.9	4.0 ± 3.2
	15	2.5 ± 0.6	2.5 ± 0.8	2.6 ± 1.1	4.1 ± 2.9
	30	2.2 ± 0.5	2.7 ± 0.7	2.5 ± 0.6	4.1 ± 3.1
Ash, g/kg	0	10.4 ± 1.5	11.2 ± 1.5	11.9 ± 1.2	8.8 ± 0.6^B^
	15	10.6 ± 1.5^ab^	11.4 ± 1.6^ab^	12.2 ± 2.2^a^	8.9 ± 0.6^Bb^
	30	12.0 ± 0.5	13.1 ± 1.7	13.1 ± 1.4	13.7 ± 4.6^A^
Ca, mg/kg	0	1.8 ± 0.1	1.7 ± 0.4	2.0 ± 0.2	2.1 ± 0.4
	15	2.1 ± 0.2	2.1 ± 0.4	2.1 ± 0.5	2.2 ± 0.4
	30	1.9 ± 0.2	1.9 ± 0.7	2.1 ± 0.2	2.4 ± 0.5
P, mg/kg	0	0.6 ± 0.2	0.6 ± 0.1	0.8 ± 0.1	0.7 ± 0.2
	15	0.8 ± 0.2	0.7 ± 0.2	0.9 ± 0.2	0.8 ± 0.1
	30	0.6 ± 0.1	0.7 ± 0.2	0.7 ± 0.1	0.8 ± 0.2
Mg, mg/kg	0	0.6 ± 0.1	0.7 ± 0.1	0.6 ± 0.2	0.6 ± 0.1
	15	0.7 ± 0.1	0.6 ± 0.1	0.6 ± 0.2	0.7 ± 0.2
	30	0.8 ± 0.1	0.6 ± 0.2	0.8 ± 0.1	0.8 ± 0.2
K, mg/kg	0	0.7 ± 0.1	0.8 ± 0.2	0.7 ± 0.1	0.7 ± 0.2
	15	0.7 ± 0.1	0.8 ± 0.1	0.8 ± 0.1	0.9 ± 0.1
	30	0.8 ± 0.2	0.9 ± 0.2	0.9 ± 0.1	0.8 ± 0.2

^a,b^Means with different superscripts in the same row are significantly different (*p* < 0.05). ^A,B^Means with different superscripts in the same column against the same variable are significantly different (*p* < 0.05). ^1^CHM-0.5 = 0.5 kg CHM; CHM-0.75 = 0.75 kg CHM; CHM-1 = 1 kg CHM.

### 3.2 Milk yield and milk composition

Milk yield of Holstein dairy cows fed different supplemental levels of CHM in early postpartum period has been presented in Table 5. Dietary supplementation of different levels of CHM had no effect on the milk yield of dairy cows at d 0, d 5, d 15, d 20, and d 25 after parturition. However, CHM-0.75 group had greater milk yield than CHM-1 group at d 10 (*p* = 0.001) and control group at d 30 (*p* = 0.040) after parturition.

**Table 5:** Milk yield (kg) of Holstein dairy cows fed different levels of dietary Chinese herbal medicine in early postpartum period.

Day	Control	CHM-0.5^1^	CHM-0.75^1^	CHM-1^1^	*p*-value
0	24.6 ± 6.3	25.0 ± 7.9	23.5 ± 7.4	21.7 ± 7.3	0.181
5	22.5 ± 6.8	25.7 ± 6.8	24.3 ± 6.7	22.9 ± 7.9	0.216
10	23.4 ± 7.7^ab^	26.0 ± 5.6^ab^	28.1 ± 7.1^a^	20.2 ± 6.6^b^	0.001
15	23.1 ± 9.9	26.2 ± 10.5	24.1 ± 12.3	23.3 ± 11.0	0.809
20	22.9 ± 8.3	28.8 ± 8.8	24.7 ± 6.5	26.4 ± 8.5	0.203
25	22.5 ± 3.8	23.1 ± 6.5	26.1 ± 7.1	22.7 ± 6.3	0.243
30	24.4 ± 4.6^b^	27.2 ± 5.3^ab^	31.6 ± 6.1^a^	28.5 ± 7.9^ab^	0.040

^a,b^Means with different superscripts in the same row are significantly different (*p* < 0.05). ^1^CHM-0.5 = 0.5 kg CHM; CHM-0.75 = 0.75 kg CHM; CHM-1 = 1 kg CHM.

Milk composition of Holstein dairy cows fed different levels of dietary CHM in early postpartum period has been depicted in Table 6. Supplemental CHM had no effect on milk fat, milk protein, lactose, and somatic cell count. Total solids were greater in CHM-0.5 group compared to other groups at d 15 (*p* < 0.05) and compared to CHM-0.75 and CHM-1 groups at d 30 (*p* < 0.05). Supplemental CHM-0.5 increased the solids not fat compared to CHM-0.75 and CHM-1 groups at d 30 (*p* < 0.05). Milk urea nitrogen was greater in control group than CHM-0.5 group (*p* < 0.05).

**Table 6:** Milk composition of Holstein dairy cows fed different levels of dietary Chinese herbal medicine in early postpartum period.

Item	Days	Control	CHM-0.5^1^	CHM-0.75^1^	CHM-1^1^
Milk fat, %	0	2.2 ± 0.2^B^	4.1 ± 2.5^B^	3.2 ± 1.4^B^	2.0 ± 0.3^B^
	15	2.1 ± 0.5^B^	4.5 ± 3.3^B^	3.2 ± 1.6^B^	2.5 ± 0.6^B^
	30	3.6 ± 1.4^A^	4.6 ± 1.1^A^	3.4 ± 1.9^A^	3.5 ± 1.2^A^
Milk protein, %	0	3.3 ± 0.3	4.5 ± 1.2	3.8 ± 0.4	3.6 ± 0.6
	15	3.6 ± 0.6	5.9 ± 3.3	3.6 ± 0.4	3.8 ± 0.5
	30	3.4 ± 0.4	4.2 ± 1.1	4.6 ± 3.1	3.5 ± 0.6
Lactose, %	0	4.3 ± 0.4	4.5 ± 0.7	4.8 ± 0.7	5.0 ± 0.2
	15	4.6 ± 0.6	4.2 ± 0.7	5.1 ± 0.5	4.9 ± 0.7
	30	4.5 ± 0.6	4.6 ± 0.3	5.0 ± 0.4	4.7 ± 0.3
Total solids, %	0	11.7 ± 0.5^b^	15.1 ± 2.1^a^	12.5 ± 1.6^b^	11.5 ± 1.0^b^
	15	11.9 ± 0.6^b^	15.8 ± 3.4^a^	12.1 ± 2.0^b^	11.6 ± 0.9^b^
	30	19.7 ± 4.1^a^	19.7 ± 2.5^a^	15.5 ± 2.9^b^	16.6 ± 3.5^b^
Solids not fat, %	0	9.1 ± 0.5	9.9 ± 1.1	9.3 ± 0.7	9.5 ± 0.6
	15	9.3 ± 0.5	11.2 ± 3.4	8.9 ± 0.8	9.4 ± 0.6
	30	10.2 ± 2.9^ab^	10.1 ± 0.9^a^	8.8 ± 0.7^b^	9.0 ± 0.5^b^
Milk urea nitrogen, mg/dL	0	16.1 ± 2.4^B^	15.8 ± 1.6	17.3 ± 3.1^B^	19.1 ± 3.3
	15	16.1 ± 3.1^B^	18.5 ± 5.7	17.6 ± 3.2^B^	18.4 ± 3.1
	30	26.9 ± 7.9^Aa^	15.9 ± 6.2^b^	21.9 ± 5.8^Aab^	20.4 ± 5.4^ab^
Somatic cell count, 10^3^/mL	0	215.2 ± 35.7	190.4 ± 29.1	207.5 ± 25.6	201.8 ± 70.4
	15	210.4 ± 34.5	189.7 ± 29.1	202.8 ± 33.6	198.6 ± 27.1
	30	196.6 ± 34.7	178.2 ± 32.3	183.2 ± 18.7	173.8 ± 20.6

^a,b^Means with different superscripts in the same row are significantly different (*p* < 0.05). ^A,B^Means with different superscripts in the same column against the same variable are significantly different (*p* < 0.05). ^1^CHM-0.5 = 0.5 kg CHM; CHM-0.75 = 0.75 kg CHM; CHM-1 = 1 kg CHM.

### 3.3 Correlation analysis of treatment and parity effects on production performance

The correlation analysis results for the impact of group, parity, lactation days, and their interactions on milk production elucidate that the treatment and parity significantly influence milk production in Holstein dairy cows during the early postpartum period. The interaction effects of treatment*parity and DIM2*parity were not statistically significant (Table 7).

**Table 7:** Correlation analysis of group, parity, lactation days and interaction on milk production of Holstein dairy cows fed different levels of dietary Chinese herbal medicine in early postpartum period.

Main effects and interactions	*χ*^2^-value	*F*-value	*p*-value
Treatment	10.64	3.55	0.014
Parity	7.96	3.98	0.019
Treatment*parity	2.67	0.53	0.750
DIM2	0.08	0.04	0.959
Treatment*DIM2	4.08	1.02	0.395
DIM2*parity	8.14	2.04	0.087
Treatment*DIM2*parity	0.19	0.10	0.908

### 3.4 Serum biochemistry

Table 8 shows the serum biochemical indices of Holstein dairy cows fed different levels of dietary CHM in early postpartum period. Serum IgG was greater in CHM-0.75 supplemented group compared to the control group at d 15 (*p* < 0.05) while, IgA and IgM remained unaffected across the groups. Serum ALT, AST, ALB, ALP, LDH, TBA, uric acid, TG, and K were not different among the groups at any observation. Serum TP was lower in CHM-0.5 group than other groups at d 15 (*p* < 0.05) and compared to CHM-0.75 group at d 30 (*p* < 0.05). Cows in control and CHM-0.75 group had greater serum GLB than those in CHM-0.5 group at d 15 (*p* < 0.05) whereas, CHM-0.75 group had greater serum GLB in comparison with CHM-1 group at d 30 (*p* < 0.05). Serum TBIL was greatest (*p* < 0.05) in CHM-0.5 and CHM-1 groups at d 15 and d 30, respectively. At d 15 and d 30, serum GGT was lowest (*p* < 0.05) in CHM-0.5 and CHM-1 groups, respectively. Cows in CHM-0.5 group had greater serum CK than CHM-0.75 and CHM-1 groups at d 15 (*p* < 0.05), whereas no difference was seen among the groups at d 30. Supplemental CHM-0.5 lowered the serum amylase at d 30 compared to control group (*p* < 0.05). At d 30, supplemental CHM-1 lowered the BUN compared to the control group (*p* < 0.05). Serum CREA was greater in CHM-0.5 group at d 15 in comparison with other groups (*p* < 0.05). Supplemental CHM-0.5 lowered the serum CHOL at d 15 (*p* < 0.05) whereas, serum CHOL was greater in control and CHM-0.75 group than other groups at d 30 (*p* < 0.05). Cows fed CHM-0.5 diets had lower serum Na levels than other groups at d 15 (*p* < 0.05), however, it was lower in all groups at d 30 except CHM-0.75 group (*p* < 0.05). Serum Cl levels were greater in CHM-0.75 group than CHM-0.5 group at d 30 (*p* < 0.05). At d 15, cows in CHM-0.5 group had lower serum Ca levels than those in other groups (*p* < 0.05) whereas, control and CHM-0.75 had greater serum Ca levels than CHM-0.5 and CHM-1 groups at d 30 (*p* < 0.05). Lower serum Mg levels were seen in CHM-1 group compared to control and CHM-0.75 groups at d 30 (*p* < 0.05). Cows in CHM-0.5 group had lowest serum P levels at d 15 in comparison with those in other groups (*p* < 0.05).

**Table 8:** Serum biochemistry of Holstein dairy cows fed different levels of dietary Chinese herbal medicine in early postpartum period.

Item^1^	Day	Control	CHM-0.5^2^	CHM-0.75^2^	CHM-1^2^
Immune function tests					
IgG, g/L	0	8.6 ± 0.7^b^	9.3 ± 1.4^ab^	11.3 ± 0.5^a^	10.1 ± 1.3^ab^
	15	9.1 ± 3.0^b^	9.9 ± 2.4^ab^	11.3 ± 2.4^a^	10.4 ± 0.6^ab^
	30	9.8 ± 0.9^b^	10.6 ± 1.5^ab^	11.6 ± 0.7^a^	10.9 ± 1.7^ab^
IgA, g/L	0	0.6 ± 0.1	0.8 ± 0.1	0.7 ± 0.1	0.6 ± 0.2
	15	0.6 ± 0.3	0.7 ± 0.2	0.8 ± 0.3	0.7 ± 0.1
	30	0.7 ± 0.1	0.8 ± 0.2	0.8 ± 0.1	0.8 ± 0.1
IgM, g/L	0	2.3 ± 0.3	2.6 ± 0.5	2.6 ± 0.2	2.4 ± 0.3
	15	2.4 ± 0.6	2.6 ± 0.5	2.8 ± 0.5	2.5 ± 0.1
	30	2.5 ± 0.5	2.8 ± 0.6	2.8 ± 0.1	2.8 ± 0.5
Liver function tests					
ALT, U/L	0	13.4 ± 7.0^B^	7.3 ± 1.7^B^	17.9 ± 7.5^B^	9.3 ± 2.1^B^
	15	23.2 ± 3.1^A^	22.4 ± 5.0^A^	27.2 ± 4.8^A^	22.4 ± 7.8^A^
	30	14.4 ± 11.0^B^	7.4 ± 2.7^B^	18.4 ± 6.8^B^	9.2 ± 2.2^B^
AST, U/L	0	44.9 ± 36.1^B^	45.8 ± 35.6^B^	44.5 ± 26.8^B^	53.5 ± 21.1^B^
	15	83.6 ± 15.9^A^	91.2 ± 17.8^A^	81.0 ± 17.6^A^	95.8 ± 31.9^A^
	30	49.8 ± 32.1^B^	51.8 ± 27.5^B^	51.8 ± 24.5^B^	62.4 ± 20.4^A^
TP, g/L	0	40.6 ± 25.6^B^	23.7 ± 5.6^B^	51.5 ± 19.8^B^	25.3 ± 5.2^B^
	15	83.2 ± 10.8^Aa^	60.1 ± 4.8^Ab^	83.0 ± 12.1^Aa^	77.1 ± 5.0^Aa^
	30	43.7 ± 23.5^Bab^	25.7 ± 7.9^Bb^	54.4 ± 16.8^Ba^	28.7 ± 4.7^Bab^
ALB, g/L	0	18.6 ± 7.6^B^	11.6 ± 4.2^B^	23.4 ± 8.7^B^	14.6 ± 3.3^B^
	15	32.2 ± 3.0^A^	34.7 ± 2.5^A^	34.5 ± 3.2^A^	35.9 ± 3.0^A^
	30	19.6 ± 8.8^B^	12.2 ± 3.9^B^	24.4 ± 7.9^B^	16.6 ± 2.9^B^
GLB, g/L	0	23.5 ± 16.7^B^	12.5 ± 7.3^B^	28.95 ± 11.9^B^	11.6 ± 2.1^B^
	15	51.0 ± 10.2^Aa^	25.4 ± 2.7^Ab^	48.5 ± 13.7^Aa^	41.1 ± 4.9^Aab^
	30	24.0 ± 15.1^Bab^	13.5 ± 4.4^Bab^	30.0 ± 10.6^Ba^	12.1 ± 2.2^Bb^
ALB:GLB	0	0.8 ± 0.1	0.8 ± 0.1^B^	0.8 ± 0.4	1.3 ± 0.2^A^
	15	0.7 ± 0.1^b^	1.4 ± 0.1^Aa^	0.8 ± 0.3^b^	0.9 ± 0.2^Bb^
	30	0.9 ± 0.2^b^	0.9 ± 0.2^Bb^	0.9 ± 0.4^b^	1.4 ± 0.2^Aa^
TBIL, μmol/L	0	0.3 ± 0.1^B^	0.3 ± 0.2^B^	0.65 ± 0.4^B^	3.4 ± 1.1^A^
	15	1.1 ± 0.6^Ab^	6.9 ± 5.4^Aa^	1.1 ± 0.5^Ab^	1.6 ± 1.1^Bb^
	30	0.4 ± 0.2^Bb^	0.4 ± 0.3^Bb^	0.7 ± 0.5^Bb^	3.5 ± 1.1^Aa^
ALP, U/L	0	24.0 ± 21.0^B^	18.8 ± 17.5^B^	40.6 ± 43.1^B^	22.8 ± 14.8^B^
	15	65.4 ± 14.6^A^	55.6 ± 10.4^A^	58.0 ± 30.1^A^	54.0 ± 5.1^A^
	30	37.0 ± 19.0^B^	20.8 ± 15.8^B^	43.6 ± 31.1^B^	25.8 ± 15.6^B^
GGT, U/L	0	18.6 ± 7.6^a^	16.7 ± 16.7^a^	17.7 ± 8.4^aB^	10.1 ± 2.8^bB^
	15	32.6 ± 8.2^Aa^	18.8 ± 5.6^b^	30.6 ± 10.1^Aa^	31.2 ± 9.9^Aa^
	30	19.2 ± 4.6^Ba^	17.6 ± 13.8^a^	19.8 ± 8.4^Ba^	9.8 ± 2.6^Bb^
LDH, U/L	0	553.2 ± 26.2^B^	479.9 ± 30.5^B^	623.7 ± 28.1	514.1 ± 20.2^B^
	15	992.2 ± 30.3^A^	753.8 ± 51.8^A^	746.0 ± 21.6	798.6 ± 68.3^A^
	30	563.2 ± 26.6^B^	498.9 ± 30.5^B^	613.7 ± 27.1	501.8 ± 18.9^B^
CK, U/L	0	130.0 ± 18.9	50.8 ± 14.2^B^	124.0 ± 17.4	93.0 ± 20.8^B^
	15	148.8 ± 40.1^ab^	211.2 ± 48.3^Aa^	123.6 ± 43.8^b^	140.0 ± 49.0^Ab^
	30	136.0 ± 10.9	54.8 ± 13.2^B^	136.0 ± 16.4	105.8 ± 19.8^B^
Pancrease function tests					
Amylase, U/L	0	26.6 ± 11.6^B^	11.1 ± 5.3^B^	22.6 ± 4.6^B^	12.0 ± 2.1^B^
	15	39.6 ± 17.6^A^	42.8 ± 9.6^A^	39.4 ± 8.2^A^	40.8 ± 22.9^A^
	30	27.6 ± 14.6^Ba^	11.4 ± 7.8^Bb^	23.8 ± 4.9^Bab^	14.8 ± 4.4^Bab^
TBA, μmol/L	0	10.3 ± 11.7	12.6 ± 4.3	9.3 ± 4.5	17.1 ± 5.3
	15	11.7 ± 4.9	20.7 ± 12.3	16.9 ± 6.7	22.1 ± 11.8
	30	13.7 ± 10.2	14.4 ± 5.3	11.1 ± 5.6	19.2 ± 5.2
GLU, μmol/L	0	2.8 ± 0.5	2.2 ± 0.4	3.3 ± 1.1	2.2 ± 0.2
	15	4.3 ± 0.2	3.8 ± 0.5	4.4 ± 0.3	4.7 ± 0.7
	30	2.9 ± 0.8	2.3 ± 0.5	3.5 ± 0.9	2.3 ± 0.3
Renal function tests					
BUN, μmol/L	0	4.1 ± 2.4	3.5 ± 1.1	4.3 ± 0.7	2.4 ± 0.5
	15	4.7 ± 0.5	3.6 ± 0.2	4.9 ± 0.5	4.7 ± 1.5
	30	4.9 ± 2.1^a^	3.6 ± 0.9^ab^	4.6 ± 0.6^ab^	2.7 ± 0.5^b^
UA μmol/L	0	29.6 ± 22.3	23.5 ± 4.5	28.6 ± 8.3	23.8 ± 0.9^B^
	15	42.4 ± 8.6	32.0 ± 14.4	41.4 ± 7.3	49.2 ± 9.3^A^
	30	32.8 ± 19.3	26.0 ± 8.6	30.6 ± 8.3	26.2 ± 3.3^B^
CREA, μmol/L	0	32.3 ± 9.4	28.2 ± 6.7^B^	35.5 ± 1.9	36.5 ± 3.7
	15	42.8 ± 3.3^b^	68.2 ± 17.9^Aa^	46.0 ± 7.4^b^	47.4 ± 7.1^b^
	30	34.2 ± 8.6	30.2 ± 5.8^B^	36.6 ± 2.9	39.0 ± 4.6
Blood lipids profile					
CHOL, mmol/L	0	2.0 ± 0.7^B^	0.7 ± 0.5	2.8 ± 1.3	0.5 ± 0.1^B^
	15	4.5 ± 1.1^Aa^	1.6 ± 0.3^b^	4.8 ± 0.6^a^	4.1 ± 2.2^Aa^
	30	2.1 ± 0.8^Ba^	0.8 ± 0.6^b^	3.1 ± 1.3^a^	0.6 ± 0.1^Bb^
TG, mmol/L	0	0.2 ± 0.1	0.1 ± 0.01	0.1 ± 0.2	0.1 ± 0.02
	15	0.3 ± 0.03	0.3 ± 0.03	0.3 ± 0.02	0.3 ± 0.1
	30	0.2 ± 0.1	0.2 ± 0.01	0.2 ± 0.1	0.2 ± 0.02
Blood minerals profile					
K, mmol/L	0	4.5 ± 0.7^B^	4.1 ± 0.3	4.5 ± 0.5	3.8 ± 0.3
	15	7.3 ± 2.5^A^	4.4 ± 0.8	6.5 ± 2.4	4.6 ± 0.6
	30	4.4 ± 0.8^B^	4.2 ± 0.4	4.7 ± 0.6	4.0 ± 0.3
Na, mmol/L	0	136.3 ± 6.4^B^	128.3 ± 3.1	137.4 ± 6.7	124.4 ± 0.6^B^
	15	151.0 ± 8.9^Aa^	131.5 ± 1.5^b^	152.5 ± 5.5^a^	146.9 ± 10.8^Aa^
	30	139.6 ± 5.7^Bb^	132.3 ± 4.5^b^	143.8 ± 8.7^a^	128.4 ± 0.6^Bb^
Cl, mmol/L	0	90.6 ± 2.2	87.2 ± 1.4	91.6 ± 1.6	93.1 ± 3.8
	15	93.6 ± 1.8	97.0 ± 9.1	94.9 ± 3.7	94.8 ± 4.1
	30	96.2 ± 2.4^ab^	92.2 ± 2.4^b^	97.5 ± 1.9^a^	97.1 ± 3.8^ab^
Ca, mmol/L	0	1.7 ± 0.3^B^	1.3 ± 0.1^B^	1.8 ± 0.2^B^	1.3 ± 0.2^B^
	15	2.3 ± 0.1^Aa^	1.9 ± 0.4^Ab^	2.2 ± 0.1^Aa^	2.3 ± 0.1^Aa^
	30	1.9 ± 0.4^Bab^	1.4 ± 0.1^Bb^	1.9 ± 0.3^Ba^	1.4 ± 0.2^Bb^
Mg, mmol/L	0	0.4 ± 0.2	0.3 ± 0.1	0.5 ± 0.2	0.1 ± 0.2^B^
	15	0.6 ± 0.2	0.6 ± 0.3	0.7 ± 0.2	0.7 ± 0.2^A^
	30	0.6 ± 0.3^a^	0.4 ± 0.1^ab^	0.6 ± 0.1^a^	0.2 ± 0.2^Bb^
P, mmol/L	0	1.2 ± 0.4	1.3 ± 0.3	1.2 ± 0.1	0.8 ± 0.2^B^
	15	1.6 ± 0.2^a^	0.9 ± 0.5^b^	1.5 ± 0.1^a^	1.8 ± 0.3^Aa^
	30	1.4 ± 0.5	1.4 ± 0.4	1.3 ± 0.2	0.9 ± 0.2^B^

^a,b^Means with different superscripts in the same row are significantly different (*p* < 0.05). ^A,B^Means with different superscripts in the same column against the same variable are significantly different (*p* < 0.05). ^1^IgG, immunoglobulin G; IgA, immunoglobulin A; IgM, immunoglobulin M; ALT, alanine transaminase; AST, aspartate transaminase; TP, total protein; ALB, albumin; GLB, globulin; TBIL, total bilirubin; ALP, alkaline phosphatase; GGT, Gamma-glutamyl transferase; LDH, lactate dehydrogenase; CK, creatine kinase; TBA, total bile acids; GLU, glucose; BUN, blood urea nitrogen; UA, uric acid; CREA, creatinine; CHOL, total cholesterol; TG, triglycerides. ^2^CHM-0.5 = 0.5 kg CHM; CHM-0.75 = 0.75 kg CHM; CHM-1 = 1 kg CHM.

### 3.5 Hematologic profile

Complete blood profile of Holstein dairy cows fed different levels of supplemental CHM are shown in Table 9. Results indicated that leukocytes (WBC), erythrocytes (RBC), hematocrit (HCT), mean corpuscular volume (MCV), mean corpuscular hemoglobin (MCH), mean corpuscular hemoglobin concentration (MCHC), erythrocyte distribution width – standard deviation (RDW-SD), erythrocyte distribution width – coefficient of variation (RDW-CV), mean platelet volume (MPV), platelet distribution width (PDW), and plateletcrit (PCT) values were not influenced by the dose of CHM. The lymphocytes range was within the range, however, their values increased in the CHM-0.5 group compared to the CHM-1 group at d 30 (*p* < 0.05). Higher granulocyte (%) values were observed in the CHM-1 group than in the CHM-0.5 group both at d 15 and d 30 (*p* < 0.05). The highest monocytes (%) level was recorded at d 30 in CHM-1 group compared to other groups (*p* < 0.05). The number of monocytes and granulocytes remained unaffected whereas, lymphocyte numbers were greater in control group than in CHM-1 group at d 15 and d 30 (*p* < 0.05). At d 30, lower hemoglobin levels were noted in the CHM-0.5 group compared to other groups (*p* < 0.05). Total platelet count increased in the CHM-1 group compared to the CHM-0.5 group at d 15 (*p* < 0.05) whereas, it was highest in control group than other groups at d 30 (*p* < 0.05). Cows in CHM-0.5 group had greater platelets than those in CHM-1 group at d 15 (*p* < 0.05), while CHM-0.75 group had greater number of platelets than CHM-0.5 and CHM-1 groups at d 30 (*p* < 0.05).

**Table 9:** Hematological profile of Holstein dairy cows fed different levels of dietary Chinese herbal medicine in early postpartum period.

Item^1^	Reference range	Days	Control	CHM-0.5^2^	CHM-0.75^2^	CHM-1^2^
Leukocytes, 10^9^/L	5.0–16.0	0	12.9 ± 5.3	13.5 ± 4.3	12.8 ± 2.9	10.7 ± 3.1
		15	14.9 ± 7.1	15.8 ± 7.4	14.3 ± 3.9	11.7 ± 3.1
		30	19.4 ± 13.9	19.2 ± 7.3	16.8 ± 6.3	16.4 ± 11.4
Lymphocyte ratio, %	20.0–60.3	0	31.3 ± 18.1	32.2 ± 19.5^B^	32.7 ± 15.7^B^	27.2 ± 15.4
		15	36.3 ± 21.2	38.5 ± 23.2^B^	33.3 ± 18.8^B^	29.1 ± 18.3
		30	44.5 ± 31.4^ab^	52.0 ± 27.1^Aa^	45.5 ± 23.9^Aab^	28.9 ± 21.4^b^
Monocyte ratio, %	4.0–12.1	0	11.2 ± 6.6^ab^	8.3 ± 4.3^a^	11.0 ± 8.5^ab^	14.4 ± 12.6^a^
		15	10.2 ± 6.9^ab^	7.4 ± 4.4^b^	9.9 ± 7.6^ab^	15.1 ± 11.6^a^
		30	8.7 ± 8.1^ab^	4.2 ± 2.4^b^	6.1 ± 3.2^ab^	10.8 ± 3.5^a^
Granulocyte ratio, %	30.0–65.0	0	51.4 ± 14.3	51.2 ± 18.4	54.0 ± 17.0	53.5 ± 16.1
		15	53.4 ± 16.6	54.2 ± 20.2^A^	56.9 ± 18.2	55.9 ± 17.1
		30	46.8 ± 26.2^b^	43.8 ± 25.1^Bb^	48.3 ± 21.1^b^	60.3 ± 18.2^a^
Lymphocytes, 10^9^/L	1.5–9.0	0	4.7 ± 5.4^B^	5.6 ± 2.1^B^	4.3 ± 2.5	3.4 ± 2.6
		15	6.6 ± 6.9^Ba^	7.2 ± 6.9^ABab^	5.0 ± 3.7^ab^	3.5 ± 2.4^b^
		30	11.9 ± 8.6^Aa^	10.9 ± 9.3^Aab^	8.8 ± 8.2^ab^	6.5 ± 7.4^b^
Monocytes, 10^9^/L	0.3–1.6	0	1.2 ± 0.4	0.5 ± 0.1	1.4 ± 1.4	1.6 ± 1.8
		15	1.2 ± 0.6	0.9 ± 0.3	1.4 ± 1.2	1.8 ± 1.7
		30	0.9 ± 0.4	0.7 ± 0.4	0.9 ± 0.4	1.5 ± 0.9
Granulocytes, 10^9^/L	2.3–9.1	0	6.97 ± 1.1	7.5 ± 2.6	6.9 ± 1.7	5.6 ± 1.6
		15	6.9 ± 1.2	7.7 ± 3.4	7.8 ± 2.5	6.4 ± 2.5
		30	6.6 ± 3.0	7.6 ± 4.5	7.1 ± 1.9	8.4 ± 3.3
Erythrocytes, 10^12^/L	5.00–10.10	0	5.6 ± 0.2	5.4 ± 0.7	5.8 ± 0.5	5.2 ± 0.3
		15	6.1 ± 0.6	5.7 ± 0.8	6.0 ± 0.6	5.6 ± 0.5
		30	66.0 ± 0.3	5.5 ± 0.6	6.3 ± 0.5	6.1 ± 0.4
Hemoglobin, g/L	90–130	0	106.7 ± 5.3	102.6 ± 13.1	105.1 ± 14.5^B^	100.3 ± 15.2^B^
		15	113.7 ± 5.6	106.6 ± 15.4	110 ± 15.6^B^	102.3 ± 16.3^B^
		30	113.6 ± 9.2^a^	97.0 ± 19.4^b^	121.8 ± 14.8^Aa^	119 ± 11.5^Aa^
Hematocrit, %	28.0–46.0	0	34.5 ± 1.6	32.9 ± 2.4	34.2 ± 5.0	32.5 ± 1.9
		15	36.7 ± 2.9	35.9 ± 4.8	36.6 ± 5.4	35.4 ± 2.7
		30	38.1 ± 3.0	32.4 ± 4.5	39.3 ± 5.4	38.7 ± 2.9
MCV, fL	38.0–53.0	0	56.7 ± 2.4	60.7 ± 2.1	53.6 ± 11.1	60.2 ± 3.5
		15	60.7 ± 3.4	62.7 ± 3.2	56.9 ± 12.1	62.3 ± 4.7
		30	62.5 ± 5.7	59.5 ± 4.4	62.7 ± 4.4	64.6 ± 3.6
MCH, pg	13.0–19.0	0	17.6 ± 1.5	14.5 ± 0.2	16.3 ± 0.7	17.1 ± 1.8
		15	18.8 ± 1.6	18.5 ± 0.6	18.1 ± 0.9	18.2 ± 1.6
		30	19.4 ± 0.7	17.5 ± 2.4	19.4 ± 0.9	18.9 ± 1.2
MCHC, g/L	300–370	0	308.3 ± 18.1	291.9 ± 6.4	278.6 ± 21.4	278.3 ± 31.3
		15	310.3 ± 21.1	295.9 ± 9.5	300.6 ± 18.4	287.3 ± 33.3
		30	312.0 ± 21.9	293.6 ± 26.7	310 ± 11.7	292.8 ± 9.8
RDW-SD, %	0.1–99.9	0	28.0 ± 3.2	29.7 ± 2.4	27.1 ± 7.2	33.7 ± 3.8
		15	31.0 ± 2.9	32.7 ± 3.9	30.1 ± 6.5	33.7 ± 3.8
		30	32.7 ± 4.1	29.7 ± 4.4	33.4 ± 3.9	34.2 ± 5.0
RDW-CV, %	14.0–19.0	0	13.4 ± 1.0	16.1 ± 1.2	16.6 ± 1.2	15.4 ± 1.2
		15	16.7 ± 1.1	17.1 ± 1.4	16.9 ± 1.4	17.4 ± 1.2
		30	17.2 ± 1.1	16.3 ± 1.7	17.5 ± 1.2	17.4 ± 1.8
Platelets, g/L	120–820	0	308.1 ± 132.7^Bb^	334.5 ± 140.2^Aab^	370.4 ± 125.6^Aa^	376.3 ± 199.1^Ba^
		15	362.1 ± 188.7^Bab^	344.5 ± 137.5^Ab^	390.4 ± 130.9^Aab^	407.3 ± 194.1^Aa^
		30	400.2 ± 160.3^Aa^	319.0 ± 143.7^Bb^	305.2 ± 162.1^Bb^	307.8 ± 159.8^Bb^
MPV, %	3.8–7.0	0	7.7 ± 1.2	8.0 ± 1.1	7.3 ± 1.0	7.1 ± 0.2
		15	8.2 ± 1.4	8.1 ± 1.3	8.0 ± 1.0	7.3 ± 0.3
		30	8.8 ± 1.9	7.8 ± 1.4	9.4 ± 1.6	7.7 ± 1.6
PDW, %	0.1–30.0	0	8.3 ± 0.6	9.0 ± 1.2	8.3 ± 1.0	7.8 ± 0.5
		15	8.5 ± 0.8	9.2 ± 1.1	8.6 ± 0.9	8.9 ± 0.8
		30	8.4 ± 0.9	8.1 ± 0.4	8.9 ± 0.7	8.8 ± 0.9
Plateletcrit, %	0.01–9.99	0	0.1 ± 0.1	0.2 ± 0.1	0.3 ± 0.1	0.2 ± 0.1
		15	0.3 ± 0.2	0.3 ± 0.1	0.4 ± 0.2	0.3 ± 0.2
		30	0.4 ± 0.4	0.2 ± 0.1	0.3 ± 0.2	0.1 ± 0.1
Platelets ratio, %	0.1–99.9	0	2.1 ± 5.6^B^	8.6 ± 2.3	6.5 ± 5.6^B^	2.3 ± 2.1
		15	4.1 ± 9.6^Bab^	8.9 ± 8.9^a^	5.7 ± 8.7^Bab^	2.5 ± 2.7^b^
		30	10.1 ± 13.9^Aab^	11.1 ± 9.2^b^	14.9 ± 10.5^Aa^	5.2 ± 6.9^b^

^a,b^Means with different superscripts in the same row are significantly different (*p* < 0.05). ^A,B^Means with different superscripts in the same column against the same variable are significantly different (*p* < 0.05). ^1^MCV, mean corpuscular volume; MCH, mean corpuscular hemoglobin; mean corpuscular hemoglobin concentration; RDW-SD, erythrocyte distribution width – standard deviation; RDW-CV, erythrocyte distribution width – coefficient of variation; MPV, mean platelet volume; PDW, platelet distribution width. ^2^CHM-0.5 = 0.5 kg CHM; CHM-0.75 = 0.75 kg CHM; CHM-1 = 1 kg CHM.

### 3.6 Reproductive performance

Reproductive performance of Holstein dairy cows fed different supplemental levels of CHM in early postpartum period has been presented in Table 10. Diameter of the first detecting follicle did not differ between the CHM-supplemented groups of cows. The CHM-0.75 group displayed a greater estrus response than a non-supplemented group of cows. Interval to estrus or ovulation after 2nd GnRH and estrus duration remained the same between the treatment groups. However, ovulation rate, follicle growth rate and ovulatory follicle diameter were significantly different in supplemented cows with a dose of 0.75 kg compared to control. Similarly, higher cows got pregnant in the CHM-0.75 group compared to the control and CHM-0.5 groups.

**Table 10:** Reproductive performance of Holstein dairy cows fed different levels of dietary Chinese herbal medicine in early postpartum period.

Item	Control	CHM-0.5^1^	CHM-0.75^1^	CHM-1^1^
Diameter of first detecting follicle (mm)	9.9 ± 3.5	8.3 ± 3.3	8.4 ± 2.8	7.8 ± 1.4
Estrus response	17/27 (63.0%)^b^	24/31 (77.4%)^ab^	26/29 (89.7%)^a^	24/30 (80.0%)^ab^
Interval to estrus after 2nd GnRH (h)	8.5 ± 0.8^b^	10.3 ± 0.6^a^	9.6 ± 0.8^a^	8.3 ± 0.8^a^
Estrus duration (h)	15.4 ± 0.6	16.5 ± 0.4	16.3 ± 0.6	15.5 ± 0.5
Ovulation rate	15/27 (55.6%)^b^	22/31 (70.9%)^ab^	24/29 (82.8%)^a^	22/30 (73.3%)^ab^
Interval to ovulation after 2nd GnRH (h)	24.5 ± 0.9	25.5 ± 0.5	25.4 ± 0.8	24.8 ± 0.8
Follicle growth rate (mm/day)	0.01^b^	0.29^b^	0.68^a^	0.31^b^
Ovulatory follicle diameter (mm)	13.9 ± 2.4^b^	15.0 ± 3.8^ab^	16.7 ± 4.0^a^	14.7 ± 2.3^ab^
Pregnancy rate	9/27 (33.3%)^b^	16/31 (51.6%)^b^	21/29 (72.4%)^a^	17/30 (56.7%)^ab^

^a,b^Means with different superscripts in the same row are significantly different (*p* < 0.05). ^1^CHM-0.5 = 0.5 kg CHM; CHM-0.75 = 0.75 kg CHM; CHM-1 = 1 kg CHM.

**Figure 2:**
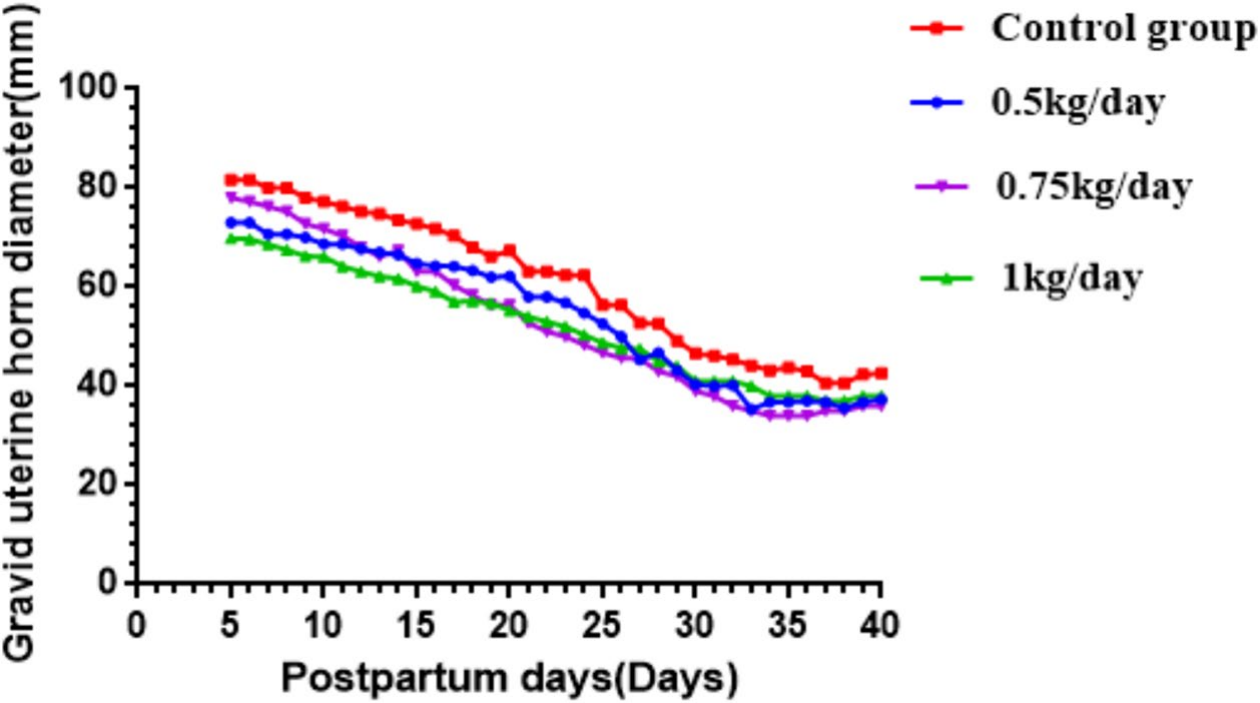
Uterine involution time and diameters of gravid uterine horn in Holstein dairy cows fed different levels of Chinese herbal medicine in early postpartum period.

The results shown in Figure 2 indicate that 30 days of supplementation of CHM during the postpartum period. The involution period of the cervix, gravid and non-gravid horns in healthy crossbred cows were 33.6 ± 2.4, 36.8 ± 3.4, and 31.6 ± 1.8 days, respectively; however, postpartum uterine infected cows had comparatively extended uterine involution period of the cervix, gravid and non-gravid horns (43.1 ± 5.2, 45.6 ± 6.8, and 39.6 ± 5.7 days, respectively).

## 4 Discussion

Over the past decades, increasing number of studies involving the use of plant-based feed additives have reported alternative solutions to antibiotics and synthetic feed additives. The variety of herbs comprising of various bioactive molecules has increased the interest to include those substances in animal diets. It has prompted the mixing and blending of different components resulting in the availability of multi-herb preparations in the market. However, this has added to the difficulty of interpreting any result due to the differences in the mechanisms as well as possible interactions among the bioactive molecules from different sources. In the present study, supplemented CHM had a variety of herbs including *E. ulmoides, A. L. (Fabaceae), C. pilosula, A. archangelica, A. amurensis G. glabra, L. striatum, W. extensa,* and *P. montana* that provided various phytogenic bioactive molecules like saponins, glycyrrhizic acid, polyphenols, polysaccharides, phytosterols, flavonoids, and carotenoids. Generally, these bioactive molecules carry out numerous beneficial activities, therefore, the use of CHM is in practice as feed additives according to “China Veterinary Drug Specifications.” Owing to the natural sources of CHM, these formulations are accepted by animals and humans with fewer drug residues and toxic/side effects. Amongst these, Eucommia, Astragalus, Codonopsis, Angelica, Atractylodes, and Liquorice nourish and strengthen the body by regulating the metabolism, and Chuanxiong promotes blood circulation, and nutrient digestion and absorption; Poria induce diuretic effects, and Pueraria act as heat dissipate and detoxifier, antibacterial and anti-inflammatory (17). Therefore, the present study was conducted to evaluate the effects of CHM on DMI, milk yield and milk composition, blood biochemistry, and reproductive performance of Holstein dairy cows in early postpartum period.

### 4.1 Dry matter intake and fecal composition

The DMI and subsequent nutrient digestibility are key components that drive the productive, reproductive, and health status of dairy cows. In this context, feed composition, minerals, and other supplemental components influence the DMI by nutritive value and palatability factors. In our study, the supplementation of CHM promoted the DMI in supplemented groups except in high-dose groups. It has been noted that CHM have poor palatability due to the presence of essential oils or a combination of cinnamaldehyde and eugenol, and increased supplementation level of CHM made the feed unpalatable because of organoleptic effects of CHM components, hence feed intake did not change in the present study. According to the Dictionary of Chinese Medicine (Nanjing University of Chinese Medicine 2006), CHM energizes the stomach and the spleen for better digestion and appetite (18). In this study, improved feed intake by cows supplemented with low or medium-dose CHM can might be attributed to the above postulation.

Cows showed an increase in ash and starch digestibility in the control and high-dose CHM groups. In contrast, higher NDF digestibility was noted in the low-dose group. Such a change in digestibility indicates the digestion stimulating activity of CHM for cellulose-degrading/fibre-degrading bacteria and probiotic bacterial growth that, in turn, maintain the pH and digestive enzyme activity (19). Previously, inhibitory CHM affected the growth of protozoa and methane-producing bacteria in the rumen (20).

An improvement was observed in daily weight gain of calves fed supplemental CHM. The improvement in the growth of calves in this study is likely linked to better nutrient utilization and the antioxidant potential of the supplements offered. Such preparations increase energy, lipid and fat metabolism when offered even to calves. Previously, better growth rates were observed in growing calves in cattle and yak (12, 21) or lambs (22, 23). The use of CHM had stimulating effects on ruminal epithelium development and ruminal microbiota in early-weaned calves.

### 4.2 Milk yield and milk composition

It is well-known that milk yield and milk composition reflect the nutritional and metabolic status, udder condition, and overall health of dairy cows. In the present study, cows fed 0.75 kg CHM per day had greater milk yield than those in control group. The used combination of CHM is an optional supplement in lactating cows. Based on the theory of traditional Chinese medicine, the CHM formulation derived from herbs (Eucommia, Astragalus, Codonopsis, Angelica, Atractylodes, Liquorice, Chuanxiong, Poria, and Pueraria) has beneficial effects (24, 25). The formulation consists of synergistic detoxification effects and promotes blood circulation of internal organs by sharing the bioactive ingredients of flavonoids or polysaccharides. Previously, various formulations of CHM were offered to cows under normal (26, 27) and stressful conditions (28, 29) that showed varying effects on milk production performance of supplemented cows.

Improved milk fat, total solids, and milk urea nitrogen levels in CHM groups suggest that the supplemental CHM improved the ruminal fermentation by promoting the ruminal microbiota for better nutrient digestion and absorption. Efficient nutrient uptake in CHM-supplemented experimental cows directed the superfluous fatty acids, carbohydrates, and amino acids towards the mammary glands for milk fat, lactose, and protein (30). In addition, the galactagogue property of different herbs (31) could be a plausible reason for quality milk production in the present study. Previously, there are variable findings in terms of milk fat, protein, lactose, milk urea nitrogen, and somatic cell count (32) that might have occurred due to the differences in experimental conditions, herb types, duration of supplementation, day in milk, and the bioavailability of active ingredients.

### 4.3 Serum biochemistry and hematological profile

Immunity-related indicators indicate the health status of animals. In the present study, the IgG levels rose in dairy cows after supplementation with a medium dose of CHM consistent with the previous studies in cows (33, 34). These studies report that CHM improved cell-mediated and humoral immunity by provoking nonspecific immunity factors via activation of the host immune cells.

Lower levels of ALT, protein, albumin, globulin, bilirubin, alkaline phosphatase, and gamma-glutamyl transpeptidase following supplementation or among the treatment groups describe the protective effects of CHM. These findings correspond to earlier documents where CHM were supplemented during stressful conditions of parturition and postpartum uterine infections (35). Beyond this, enormous fluctuations in protein and liver enzyme levels could indicate liver cell damage and increased liver cell membrane permeability, subsequently causing leakage of enzymes into the blood. Whereas the fluctuations in liver function tests are also linked to negative energy balance during the early postpartum time in cows due to excessive depletion of body fat to fulfill the energy for milk production (36). In the present study, no change was observed in total bile acids and glucose levels, which indicates that CHM provision did not influence insulin secretion Low amylase levels after CHM supplementation indicate the increased utilization of starch in the small intestine by amylase thereby reflecting the reduced amylase level. The CHM contributes the various nutritional factors, i.e., glucose, amino acids, fatty acids, and others that are used for synthesis and stimulating different enzymes, including amylase. In the present study, creatinine, urea and uric acid were within reference range after CHM supplementation suggesting the normal functioning of renal system. A decreasing trend was observed in renal function tests in CHM-supplemented groups but was not always significant. Similar findings were observed earlier in ruminants (34). Increased serum urea and creatinine levels reflect the protein intake and digestibility, fatty acids breakdown by the liver, muscular breakdown, and ruminal urea recycling (33). Serum lipids, total cholesterol, and triglycerides are the main lipids class particularly influenced by dietary factors. In the present study, supplemental CHM influenced the total cholesterol but not triglycerides. Reduced serum total cholesterol levels by CHM supplementation indicate the emulsifying effects (37) and fibrous nature (38). Such dietary properties regulate the ruminal microbiota, digestion, and absorption of nutrients.

The macro- and mirco-elements are essential in animals for normal physiological and biochemical processes and milk and meat production in ruminants. The mineral contents varied during the first and second observations after CHM provision might be related to the lactation stage of cows (39). Reported higher mineral levels by adding a CHM mixture to the diets of goats. They described that the CHM mixture remained unaffected by ruminal action and was transported to the intestinal region, where it positively influenced the hindgut digestion of nutrients.

No earlier studies have reported the effect of supplemental CHM on the hematological profile of cows. In the present study, although there were variations in many hematological variables between the supplemented and control groups, they existed in normal ranges. Thus, CHM supplementation to cows at different levels had no negative effects on hematology. These findings are consistent with previous studies reporting the effects of different herbs on hematology (34, 40).

### 4.4 Reproductive performance

In order to avoid the prepartum and postpartum reproductive disorders, management of dairy cows may be subjected to nutritional and hormonal regimens before and after calving to support the early uterine involution and resumption of ovarian activity. Our study demonstrated that CHM supplementation can improve the estrus, ovulation, and conception rates in synchronized dairy cows in addition to expediting the uterine involution in early postpartum period. Consistent with our findings, previous studies have reported the earlier resumption of postpartum reproductive events in cows fed diets supplemented with CHM (32–34). In addition, improvements were witnessed in cows suffering from calving or postpartum reproductive disorders in response to dietary CHM supplementation (30, 33). The exact mechanism by which dietary CHM supplementation improved the reproductive performance of cows in early postpartum period is not yet known. It is speculated that the improvement in DMI providing superfluous energy might have supported the occurrence of these ovarian activities due to the potential antimicrobial, immunomodulatory, antioxidative, and energy and gut ecosystem enhancing properties of CHM. Increased permeability of blood vessels in addition to lowered blood triglycerides and cholesterol might have improved the uterine health in cows fed dietary CHM. Earlier studies have provided preliminary evidence regarding the positive effect of CHM on the fertility of cows in early postpartum period. It is, however, necessary to establish the efficacy, safety margins, and mechanisms of CHM in improving the fertility of cow using well-controlled experiments with larger sample size.

## 5 Conclusion

In conclusion, the CHM preparation had no adverse effects on the biochemical indicators for immunity, digestibility, metabolism, and production performance of cows. The estrus, ovulation, and pregnancy rates were improved by CHM supplementation prior to synchronization of dairy cows. No significant change in uterine involution was observed in uterine infected or healthy CHM-supplemented cows. However, there are certain limitations of the present study. The period of this study has focused on the early postpartum period (early lactation) that requires further evaluation of supplemental CHM in mid and late lactation. Moreover, this study lacks the investigation of mechanisms by which supplemental CHM exerted these effects on the studied biological indicators. Further studies are suggested to elucidate the mechanisms and pathways involved in the beneficial effects of CHM in dairy cows.

## Data availability statement

The original contributions presented in the study are included in the article/supplementary material, further inquiries can be directed to the corresponding author.

## Ethics statement

Before execution of the study, an approval was obtained from the Animal Experimental Ethical Inspection of Laboratory Animal Centre, Huazhong Agricultural University, Wuhan (HZAUCA-2018-004). All the experimental protocols were performed according to the guidelines of the Committee of Animal Research Institute of the university. The studies were conducted in accordance with the local legislation and institutional requirements. Written informed consent was obtained from the owners for the participation of their animals in this study.

## Author contributions

AA: Conceptualization, Data curation, Formal analysis, Methodology, Resources, Software, Validation, Visualization, Writing – original draft, Writing – review & editing. UA: Conceptualization, Data curation, Formal analysis, Supervision, Writing – review & editing. ZN: Conceptualization, Data curation, Formal analysis, Methodology, Resources, Software, Validation, Writing – review & editing. ZA: Conceptualization, Data curation, Methodology, Project administration, Resources, Software, Writing – review & editing. WL: Conceptualization, Data curation, Formal analysis, Investigation, Project administration, Resources, Writing – review & editing. CR: Conceptualization, Formal analysis, Investigation, Methodology, Validation, Writing – review & editing. XP: Conceptualization, Data curation, Formal analysis, Funding acquisition, Investigation, Methodology, Resources, Software, Supervision, Visualization, Writing – review & editing. SW: Conceptualization, Formal analysis, Funding acquisition, Investigation, Methodology, Resources, Software, Supervision, Validation, Visualization, Writing – review & editing.
